# Actin Filaments at the Leading Edge of Cancer Cells Are Characterized by a High Mobile Fraction and Turnover Regulation by Profilin I

**DOI:** 10.1371/journal.pone.0085817

**Published:** 2014-01-17

**Authors:** Gisela Lorente, Emilio Syriani, Miguel Morales

**Affiliations:** 1 Neurophysiology Laboratory, Deptartment of Physiological Sciences I, School of Medicine, University of Barcelona, Barcelona, Spain; 2 Structural Synaptic Plasticity Lab, Department of Neurodegenerative Diseases, CIBIR Piqueras 98, Logroño, La Rioja, Spain; University of Illinois at Chicago, United States of America

## Abstract

Cellular motility is the basis for cancer cell invasion and metastasis. In the case of breast cancer, the most common type of cancer among women, metastasis represents the most devastating stage of the disease. The central role of cellular motility in cancer development emphasizes the importance of understanding the specific mechanisms involved in this process. In this context, tumor development and metastasis would be the consequence of a loss or defect of the mechanisms that control cytoskeletal remodeling. Profilin I belongs to a family of small actin binding proteins that are thought to assist in actin filament elongation at the leading edge of migrating cells. Traditionally, Profilin I has been considered to be an essential control element for actin polymerization and cell migration. Expression of Profilin I is down-regulated in breast and various other cancer cells. In MDA-MB-231 cells, a breast cancer cell line, further inhibition of Profilin I expression promotes hypermotility and metastatic spread, a finding that contrasts with the proposed role of Profilin in enhancing polymerization. In this report, we have taken advantage of the fluorescence recovery after photobleaching (FRAP) of GFP-actin to quantify and compare actin dynamics at the leading edge level in both cancer and non-cancer cell models. Our results suggest that (i) a high level of actin dynamics (i.e., a large mobile fraction of actin filaments and a fast turnover) is a common characteristic of some cancer cells; (ii) actin polymerization shows a high degree of independence from the presence of extracellular growth factors; and (iii) our results also corroborate the role of Profilin I in regulating actin polymerization, as raising the intracellular levels of Profilin I decreased the mobile fraction ratio of actin filaments and slowed their polymerization rate; furthermore, increased Profilin levels also led to reduced individual cell velocity and directionality.

## Introduction

Cellular motility is a complex process that occurs in all cell types [1]. Migration over a flat surface involves the protrusion of a thin membrane mantle, the lamella, filled with an intricate actin branched network. The force for the membrane protrusion and extension is provided by controlled and restricted actin polymerization at the closest edge of the membrane, the so-called leading edge. During elongation, actin filaments are polarized with their barbed end (or plus end) pointing towards the membrane [2], which is pushed by the filaments, forcing the extension of the lamella. The lamella extension, therefore, is what determines the directionality and movement of the cell [3]. Close regulation of cell migration is essential for development, wound healing and immune responses, whereas aberrant and uncontrolled cell motility is a recurrent feature in several types of cancer cells.

A number of studies indicate that Profilin I (PfnI), an essential actin-binding protein, may play an important regulatory role in the process of cellular motility. Thus, *Dictyostelium amoebae* mutants for PfnI exhibit motility and cytokinesis defects [4], as does “chickadee”, the null mutant for the homolog of PfnI in *Drosophila* [5]. Likewise, silencing PfnI in human vascular endothelial cells induces inhibition of motility and defects in membrane protrusion [6]. Furthermore, PfnI knockout has been shown to result in early embryonic cell death in mice [7].

Of the members of the Profilin family (PfnI to Pfn IV), PfnI is the most widely expressed. It was originally identified as a G-actin sequestering protein [8], and since then, it has been assigned several other functions, including the nuclear–cytoplasm traffic of actin by binding to exportin 6 [9], mRNA splicing [10], as well as vesicular endocytosis by interacting with clathrin and valosin-containing protein [11]. Nowadays, it is commonly accepted that its main role is to promote actin polymerization by catalyzing the exchange of ADP for ATP on the G-actin monomer [12]. Furthermore, the structure of PfnI contains one polyproline binding domain (PLP) [13], [14] and two phosphoinositide binding sites [15]–[17]. By virtue of the former, PfnI interacts with a profusion of proline-rich proteins. Among others, it directly binds to Ena/VASP [18], N-WASP [19], WAVE [20] and the actin nucleator family of formins [21]. All of these proteins recruit PfnI to the zones of dynamic actin remodeling, such as the leading edge of the lamellipodia. The intracellular localization of PfnI confirms its association with areas of intense actin polymerization, and thus PfnI is found in PTk2 microfilaments of marsupial fibroblasts [22], leech neuronal growth cones [23], Bovine Trabecular Meshwork lamellas [24], protruding areas of rat fibroblast [25], and near the advancing edge of endothelial cells [26].

A fundamental characteristic of tumor cell invasion and metastasis is an increased motility and migration capacity of the cells. Interestingly, the expression levels of PfnI are down-regulated in several invasive breast, pancreatic and hepatic cancer cell types [27]–[31]. Further reduction of PfnI levels by silencing its expression results in even higher motility [29], [32] and tumor progression [33]. The opposite is also true: an increase in PfnI levels reduces cell invasiveness and motility, followed by an up-regulation of stress-fibers and focal adhesion [27], [29]. Moreover, PfnI overexpression suppresses both ectopic and orthotopic tumorigenicity and micro-metastasis [32]. For tumor suppression activity to occur via this mechanism, both the actin and phosphoinositide binding sites in PfnI must be functional [30], [32], [34]. Furthermore, PfnI overexpression has been reported to up-regulate PTEN expression, therefore indirectly suppressing Akt activity by controlling phosphoinositide production [35].

The phosphoinositide binding of PfnI is more important than previously expected. The Ena/VASP family of proteins uncaps the free barbed ends of actin, allowing their progressive elongation [36]. Lamellipodin (Lpd) binds Ena/VASP through an EVH1 domain, and specifically interact with PI(3,4)P_2_. In this way, the membrane target of Lpd is capable of recruiting Ena/VASP to the membrane. A series of recent findings suggest that PfnI restricts the available pool of PI(3,4)P_2_. In this way, PfnI would negatively regulate phosphoinositide levels at the membrane, and indirectly limit Lpd-Ena/VASP targeting to the membrane [37]. In MDA-MB-231 cells, PfnI depletion regulates Lpd accumulation, indirectly raising Ena/VASP concentration at the leading edge, and consequently promoting actin polymerization and the reported hypermotility after PfnI depletion [38]. However the underlying consequences of these PfnI suppressive actions on actin turnover remain to be elucidated. Experimental evidence indicates that a functional actin binding domain of PfnI is crucial to reducing cancer cell motility and tumor phenotype [29], [30]. Given the plethora of experimental evidence supporting the importance of PfnI in regulating actin polymerization and cell migration, it is puzzling how low expression levels of PfnI are associated with enhanced cellular motility, and how actin treadmilling can be regulated.

In this report, the turnover of lamellipodial actin was examined using Fluorescence Recovery After Photobleaching (FRAP) [39]. Our results indicate that the cytoskeleton at the leading edge of cancer cells is much more dynamic than that of non-tumor cells. Moreover, increasing PfnI levels in cancer cells leads to reduced actin treadmilling and impaired cellular motility. Finally, we also found evidence suggesting that actin treadmilling in cancer cells is insensitive to extracellular regulation by growth factors.

## Materials and Methods

### Cell cultures

MDA-MB-231 human breast tumor cell lines were cultured in DMEM F12-Ham medium and 10% FBS (Sigma). The MCF10A human mammary epithelial cell line was grown in the following culture medium: HEPES 15 mM, horse serum 5%, EGF 20 ng/ml, Hydrocortisone 0.5 mg/ml, cholera toxin 100 ng/ml, human insulin 10 mg/ml, 50 mg/ml penicillin and 50 U/ml streptomycin in DMEM F12 HAM (all supplied by Sigma). The A549 human lung tumor cell line, MEF mouse embryonic fibroblasts and HeLa human cervix tumor cell line were cultured in DMEM, supplemented with 50 mg/ml penicillin, 50 U/ml streptomycin and 10% FBS (Sigma Aldrich). MDA-MB-231 cells were purchased from ATTC (USA; Ref. HTB-26). MCF10A cells, passage 8, were a generous gift from LP Saucedo (CNIO, Madrid, Spain). A549 and HeLa cells, passage 10, were a generous gift form H. Aguilar (ICO, Barcelona, Spain). MEF cells, passage 6, were generous gift from A. Angulo (IDIBAPS, Barcelona).

### Immunocytochemistry and image analysis

Briefly, for immunocytochemical analyses, cell cultures were rinsed in PBS and fixed for 15 min in 4% paraformaldehyde/PBS. Coverslips were then washed three times in PBS and incubated for 30 min in blocking solution (2% goat serum, 2% serum albumin, 0.1% Triton X-100 in PBS). Antibodies were diluted to the appropriate concentration in blocking solution. Coverslips were incubated for 60 min in the antibody solution. The samples were subsequently washed three times and incubated for 30 min with the appropriate fluorescence-conjugated secondary antibodies. Finally, coverslips were washed five times and mounted with Mowiol. Fluorescence images were obtained with an inverted microscope (Olympus IX70), using a TILL monochromator as light source. Pictures were taken with an attached cooled CCD camera (Orca II-ER, Hamamatsu).

The following antibodies were used: anti-vinculin monoclonal antibody (Chemicon, ref. MAB3574) and anti-Profilin polyclonal and monoclonal antibodies (Cell Signaling, ref. 3237 and Synaptic system, ref. 308 011). Secondary antibodies, Oregon green Phalloidin and Phalloidin-Alexa Fluor® 594 were purchased from Invitrogen. Image J analysis software (National Institutes of Health) was used to quantify spreading and focal adhesion.

### Cell area measurements and Focal adhesions quantification

Briefly, up to 10000 cells were seeded on 12 mm coverslips in 24 wells plates. After the corresponding treatment, the cells were fixed in 4% PFA and stained with Oregon–green Phalloidin or Phalloidin-Alexa Fluor® 594. Despite the lack of stress fibers, Phalloidin staining enabled the measurement of the whole cell surface. Cell area was quantified by digital threshold analysis using Image J software.

### Recombinant protein

In some experiments, the intracellular levels of Profilin were modified using a membrane permeable version of PfnI [24], [40]. PTD4-Profilin (21 kDa) was generated by fusing a transduction domain, PTD4 [41], to Profilin. The recombinant protein was expressed in *E. Coli* and purified as described previously [40]. Briefly, competent *E. Coli* BL21-PLys transformed with the pRSETA-PTD4-Pfn I vector were induced by adding 1 mM IPTG (Sigma) at 37°C for 6 h. Bacterial pellets were lysated by freezing and thawing protocol in liquid N_2_, followed by sonication on ice in the presence of DNAse and a protein inhibitor cocktail (Sigma). Cellular lysates were resolved by centrifugation, and the soluble protein was isolated by employing Ni-NTA resin-packed columns (Quiagen). Protein wash and elution was carried out with high concentrations of imidazole. Buffer exchange and concentration of the recombinant protein were performed by centrifugation in Amicon Ultra-15 10000 MWCO centrifugal filters (Millipore), replacing the elution media with PBS. Proteins were frozen in liquid N_2_ and stored at −80°C in 10–15% glycerol-PBS. Bacteria and proteins were handled according to the Safety Guidelines for Laboratory Personnel Working with Trans-Activating Transduction (TAT) Protein Transduction Domains.

### Transfection

Transfection was performed using the Efectene Transfection Reagent kit from Qiagen, following the manufacturer's instructions. Several expression vectors were used: CMV-GFP, CMV-GFP-actin and CMV-MembraneCherry kindly provided by Dr. F Tebar (University of Barcelona, Spain), and CMV-GFP-PfnI kindly provided by Dr. Hitomi Mimuro (University of Tokyo, Japan).

### Stable cell lines

Stable cell lines were generated from MDA-MB-231 cancer cell line transfected with plasmids expressing GFP-actin, GFP-PfnI, MembraneCherry-PfnI and GFP under a CMV promoter control (all work was performed with cell passages from 6 to 8). Transfections were performed with Efectene Transfection Reagent (Qiagen), as described in the protocol. Transfected cells were selected with three weeks incubation on gentamicin 1 mg/ml (Sigma). FACS was used to separate high- and low-expressing GFP-actin cells. The buffer cell sorting solution used consisted of 5 mM EDTA, 25 mM HEPES pH 7.0, 1% FBS (heat-inactivated) and PBS without Ca^2+^/Mg^2+^. Relative levels of recombinant protein expression were analyzed by Western blot.

### Cell motility assay

MDA-MB-231 cells were seeded on 6-well plates (Nunc) at 40% confluence and labeled with Draq5, a cell-permeable far-red fluorescent DNA dye, for 5 min (Cell Signaling Technology). Culture plates were mounted on the stage of a Leica DMITCS SL laser scanning confocal spectral microscope (Leica Microsystems Heidelberg, GmbH) attached to a Leica DMIRE2 inverted microscope equipped with an incubation system with temperature and CO_2_ control. All experiments were performed at 35°C and 5% CO_2_. For visualization of Draq5 stained nuclei, images were acquired using a 40× objective lens (NA 1.32) and excited with a 633 nm laser line. The confocal pinhole was set at 4.94 Airy units to minimize fluorescence loss. Pictures were taken every 5 min for 8 h. Image J analysis software (NIH) was used for velocity and directionality quantification. Directionality data was presented as the linear distance (End) divided by overall distance length during 8 h, as described by Pankov et al., 2005 [42].

### Fluorescence recovery after photobleaching (FRAP)

FRAP experiments were performed using the following protocol∶10 single scans (pre-bleaching) were acquired at 300 ms intervals, followed by 20 bleach scans at full laser power using a square area of 24 µm^2^. During the post-bleaching period, 30–60 scans were acquired at 300 ms intervals, followed by 100 images acquired at 5 s intervals, this period of time was necessary to allow the fluorescence to reach equilibrium. In order to resolve the initial fast recovery, some experiments were performed using the Leica fly mode acquisition; bleaching was performed during the X fly forward scan using 100% laser power, and during backward scan, fluorescence was read with laser intensity set to imaging values (185 ms images interval), while post-bleaching images (30–60 s) were acquired at the same time interval. To avoid significant photobleaching, the excitation intensity was attenuated to ∼5% of the laser power during image acquisition. Fluorescence recovery was quantified using Image Processing Leica Confocal Software. Background fluorescence was measured in a random field outside the cell and subtracted from all measurements. The fluorescence signal measured in the region of interest (ROI), was corrected for acquisition photobleaching and fluctuations of whole fluorescence following a double normalization method, determined as follows: Irel = It/I0 * T0/Tt, where It is the average intensity in ROI at time t; I0 is the average intensity of the ROI during the pre-bleaching period; T0 is the intensity during pre-bleaching of the non-bleached area (normally, a neighboring cell or the nuclear region); and Tt is the intensity at time t of this area. The introduction of the correction factor (T0/Tt) accounts for possible small fluctuations in total fluorescence intensity caused by the bleach itself, and yields a more accurate estimate of the measured fluorescence in the ROI [43], [44].

The net fluorescence recovery (mobile fraction; Mf) measured in the region of interest was determined as: Mf  =  Fend-Fpost/Fpre-Fpost, where Fend is ROI mean intensity at the steady-state; Fpost represents ROI intensity after photobleaching; and Fpre is the mean pre-bleaching ROI intensity. The recovery time constant (τ) was obtained by curve fitting using Prism Software (GraphPad), assuming a one-exponential model (bottom, then increasing to the top).

### Intracellular amounts of recombinant Profilin

Calculation of intracellular amounts of PTD4-PfnI was done by Western blot densitometry. Briefly, cells growing in 25 cm^2^ flasks were washed five times, trypsinized, and thoroughly washed by centrifugation to reduce the extracellular protein concentration. Cell lysates were run in a SDS-polyacrylamide gel. Diluted amounts of purified recombinant PTD4-PfnI, were loaded in the same wells. Both proteins, endogenous Profilin and PTD4-PfnI were easily distinguished by their molecular weights and were probed with a monoclonal antibody against Profilin I.

### Statistics

All data are represented as means ± SEM and were analyzed using Prism software (version 4.0 and 6.0, Graphpad). The data was analyzed using the Student's t-test when appropriate. Significance levels were noted as follows: *p<0.05, **p<0.01, and ***p<0.001.

## Results

### Actin dynamics of cancer cells are characterized by a high mobile fraction and short recovery time

In order to study the dynamics of the actin cytoskeleton at the growing leading edge of tumor cells, we have chosen the MDA-MB-231 breast cancer cell line. This cell line carries the *KRAS* G13D and the *BRAF* G464V mutations [45] and it is particularly suitable for pre-clinical studies, as it is highly aggressive, both *in vitro* and *in vivo* [46]. MDA-MB-231 cells in culture exhibit a continuous and high-motility lamella extension, presenting numerous ruffles along the membrane, which makes this cell type an excellent candidate to study the turnover of lamellipodial actin using FRAP. In order to facilitate the imaging experiments, we had previously generated a stable transfected MDA-MB-231 cell line expressing the GFP-actin construction. The relative level of GFP-actin expression was examined by Western-blot and compared with GFP expressed cell line as a control, on average, the expression of the fluorescence protein resulted in 5.2% of the total Profilin (data not shown).

**Figure 1:**
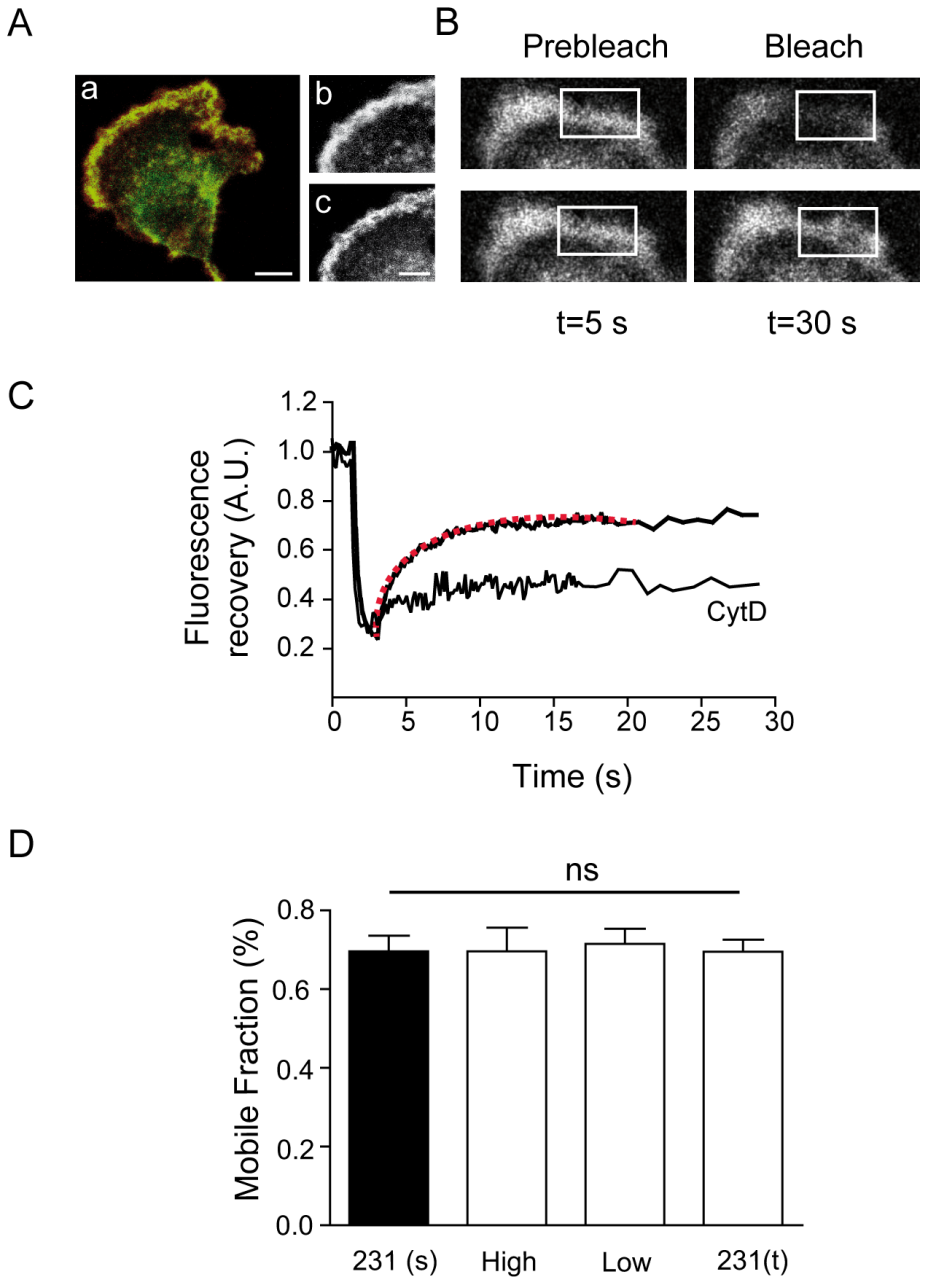
The actin cytoskeleton at the leading edge of MDA-MB-231 is characterized by a high mobile fraction and dynamics. A) MDA-MB-231 cells transfected with GFP-actin, double stained with Phalloidin-Alexa 594 (a). Actin fluorescence accumulates mainly at the leading edge area. Scale bar 5 µm. Notice that MDA-MB-231 cells have a conspicuous lack of actin stress fibers. Rigth: Detailed section of the leading edge, showing the accumulation of GFP-actin (b) and actin filaments (c). Scale bar 2 µm. B) Representative picture frames from sequential FRAP experiments. Before FRAP (pre-bleaching), after high-intensity laser exposure (bleaching), during the initial phase of recovery (5 s) and at the end of the stable part of the curve (30 s). The bleached leading edge area is indicated by the white box (4×6 µm). C) Example of a FRAP experiment; fluorescence recovers to an average value of 71.5±4% following a monoexponential time course (fit depicted by a red dotted line). Incubation with cytochalasin D (90 nM) inhibits fluorescence recovery (CytD). D) Summary graph of the mean mobile fractions from MDA-MB-231 cells. Stable transfected cells (s, n = 44), high GFP-actin expressers (high, n = 10), low GFP-actin expressers (low, n = 10) and transiently transfected cells (231t, n = 6). Mean values of mobile fraction do not show statistical differences under any experimental conditions when compared with the stable transfected cell line (Student's t-test).

The characteristic leading edge of MDA-MB-231 cells is a 2 µm broad structure enriched in GFP-actin and easily identified by Phalloidin-Alexa 594 staining (Figure 1A). A rectangular area large enough to cover the entire leading edge (4 µm wide and 6 µm long) was photobleached [39] (Figure 1B). As shown in Figure 1C, MDA-MB-231 cells exhibited a recovery of the fluorescence close to a 70% after 15 s. The recovery kinetics was described by a two-component exponential curve evidencing an initial fast component followed by a second slower component. The initial component had a recovery time of close to 500 ms, similar to the value obtained when the recovery was examined in monomeric GFP-transfected cells (tau 100–200 ms; Figure S1). This is most likely due to the rapid diffusion of GFP-actin monomers. The initial rapid component was only detected when a fast acquisition protocol was employed, and was therefore neglected in most of the experiments. The second slower component was driven by actin polymerization [47], [48], which was confirmed by the addition of 90 nM cytochalasin D (a reversible barbed–end blocker) to the culture media five minutes prior to the FRAP experiment. In the presence of cytochalasin D, GFP-actin fluorescence failed to recover (Figure 1C), confirming that actin polymerization drives the recovery of fluorescence. On average, the mobile fraction measured 30 seconds after the time of maximum bleaching was estimated to be 71.5±4%; this second component was fitted by a one-exponential curve with a mean tau value of 4±0.3 seconds (red dotted line in Figure 1C).

We then asked whether the observed mobile fraction values were dependent on GFP-actin expression levels. To this end, we analyzed recovery levels for three different conditions: cells with high or low GFP-actin expression levels (populations sorted by flow cytometry from the generated stable GFP-actin cell line) and after a transient transfection for 48 hours. The results, summarized in Figure 1D, indicated that the mean mobile fraction was similar for all three conditions, supporting the hypothesis that the actin recovery rate was not dependent on intracellular GFP-actin concentration.

**Figure 2:**
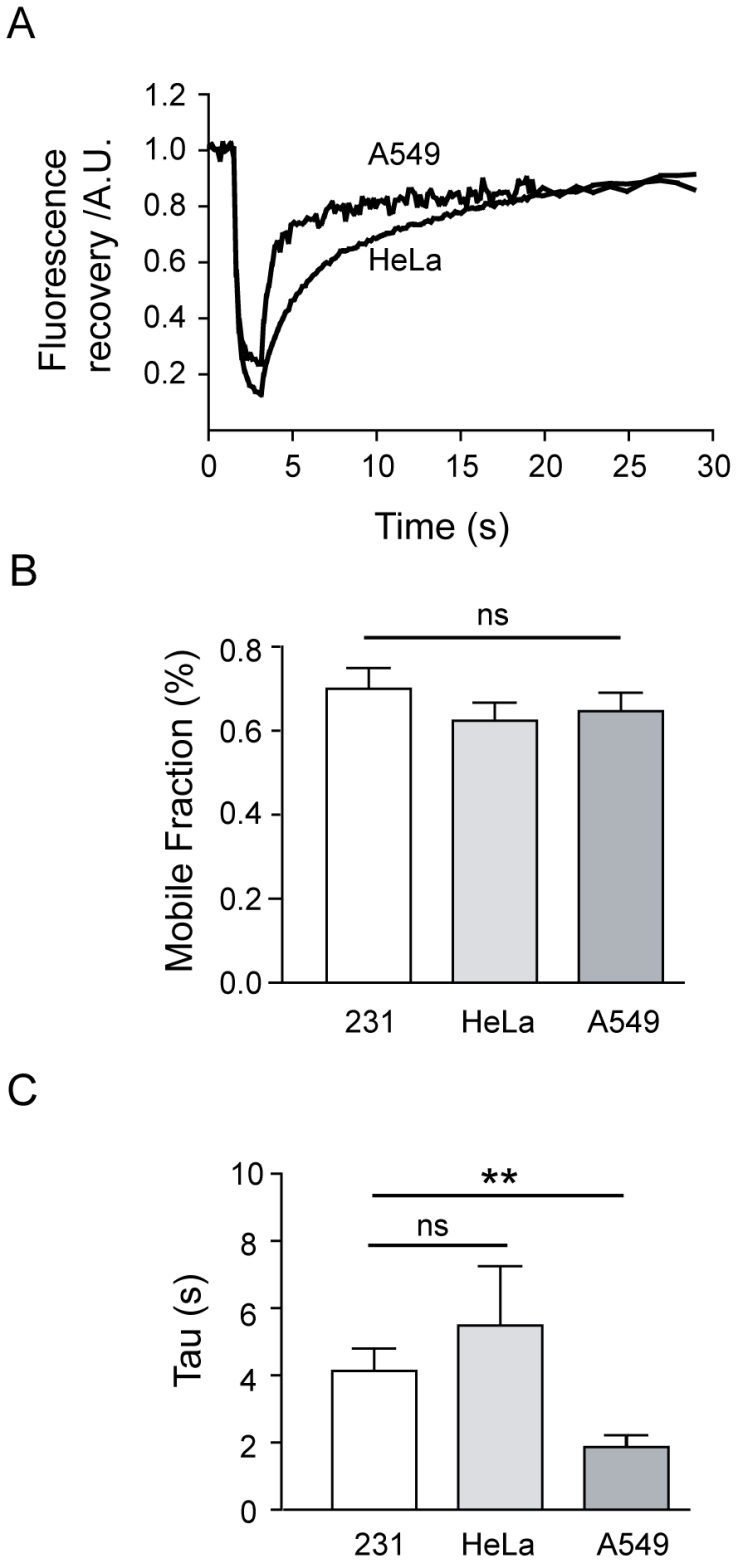
A highly dynamic cytoskeleton is a characteristic of tumor cell lines. A) Two examples of individual fluorescence recovery of GFP-actin obtained from A549 and HeLa cells. B) Bar graph of the average mobile fraction comparing MDA-MB-231, HeLa and A549 cells. The calculated mean values were 62.4±4.2% for HeLa (n = 12) and 64.7±4.3% for A549 (n = 10). No statistical differences were found when compared with MDA-MB-231 cells. C) Mean values of the tau exponential values obtained from the recovery curves fitting. MDA-MB-231 cells recovered with a tau value of 4.1±0.6 s, while HeLa cells displayed a tau of 5.5±1.7 s. A549 cells showed the fastest recovery of all, with a tau of 1.9±0.35 s, which was statistically different from the MDA-MB-231 recovery time (p<0.005, Student's t-test).

To explore whether this range of actin recovery is specific to the MDA-MB-231 cells or is a common characteristic of most cancers of epithelial origin, two additional cancer cell lines were studied. Thus, actin dynamics at the leading edge of HeLa and A549 cells, a cervix and a human pulmonary adenocarcinoma respectively [49], [50], were examined after transient transfection with GFP-actin (Figure 2A). Both cell lines exhibited a large mobile fraction after 30 seconds, with mean values similar to those of MDA-MB-231 cells (values from 62 to 64%; Figure 2B) and recovery times varying within a similar range, from 2 to 5 seconds (Figure 2C). Incidentally, A549 cells exhibited the fastest recovery, with a tau value close to 2 seconds (Figure 2C).

**Figure 3:**
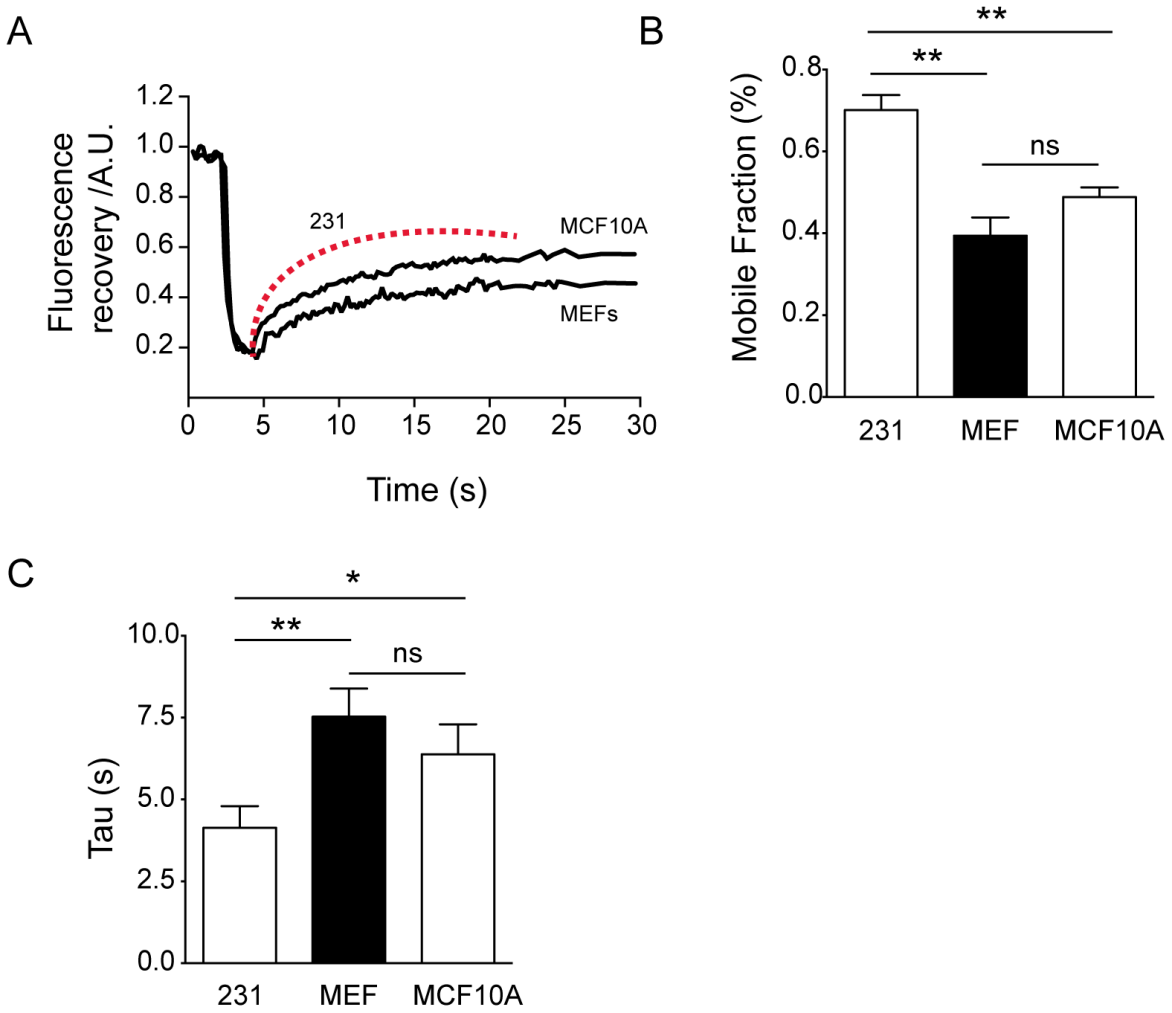
Non-tumor cells display a lower mobile fraction and slower recovery. A) Two examples of individual actin recovery curves at the leading edge after photobleaching of GFP-actin from MCF10A (human) and MEF (murine) cells. The recovery of MDA-MB-231 cells is plotted as a red dotted line for comparison. B) Summarized data of the mobile fractions of MEFs (39±4.2%, n = 18) and MCF10A (51.7±1%, n = 6) compared to MDA-MB-231 tumor cells. Each cell type shows statistical differences when compared with the MDA-MB-231 mobile fraction recovery (p<0.005, Student's t-test). The mobile fractions of MCF10A and MEF cells were not statistically different (Student's t-test). C) The recovery times of both MEFs (tau = 7.5±1.6 s) and MCF10A cells (tau = 6.5±0.4 s) were statistically different than that of MDA-MB-231 cells (p<0.005 and p<0.05; Student's t-test).

Since MDA-MB-231 breast cancer cells have an epithelial origin, we also compared their actin dynamics with that of a non-cancer cell line of MCF10A, a human epithelial mammary cell line transfected with GFP-actin. The results (summarized in Figure 3) indicated that the cytoskeleton of MCF10A had a lower actin mobile fraction than that of the counterpart cancer cell line (50% versus 70%: Figure 3A–B). Recovery time was also different, with an average value close to 7 seconds. Similar results were obtained when fibroblast cells of a non-related origin were examined. Murine Embryonic Fibroblasts (MEFs) had a mobile fraction value close to 40%, with a recovery time of around 8 seconds (Figure 3A–C). In summary, the experimental data indicate that cancer cells tested show a much more dynamic cytoskeleton than non-cancer cells.

### MDA-MB-231 actin dynamics are independent of the presence of extracellular growth factors

**Figure 4:**
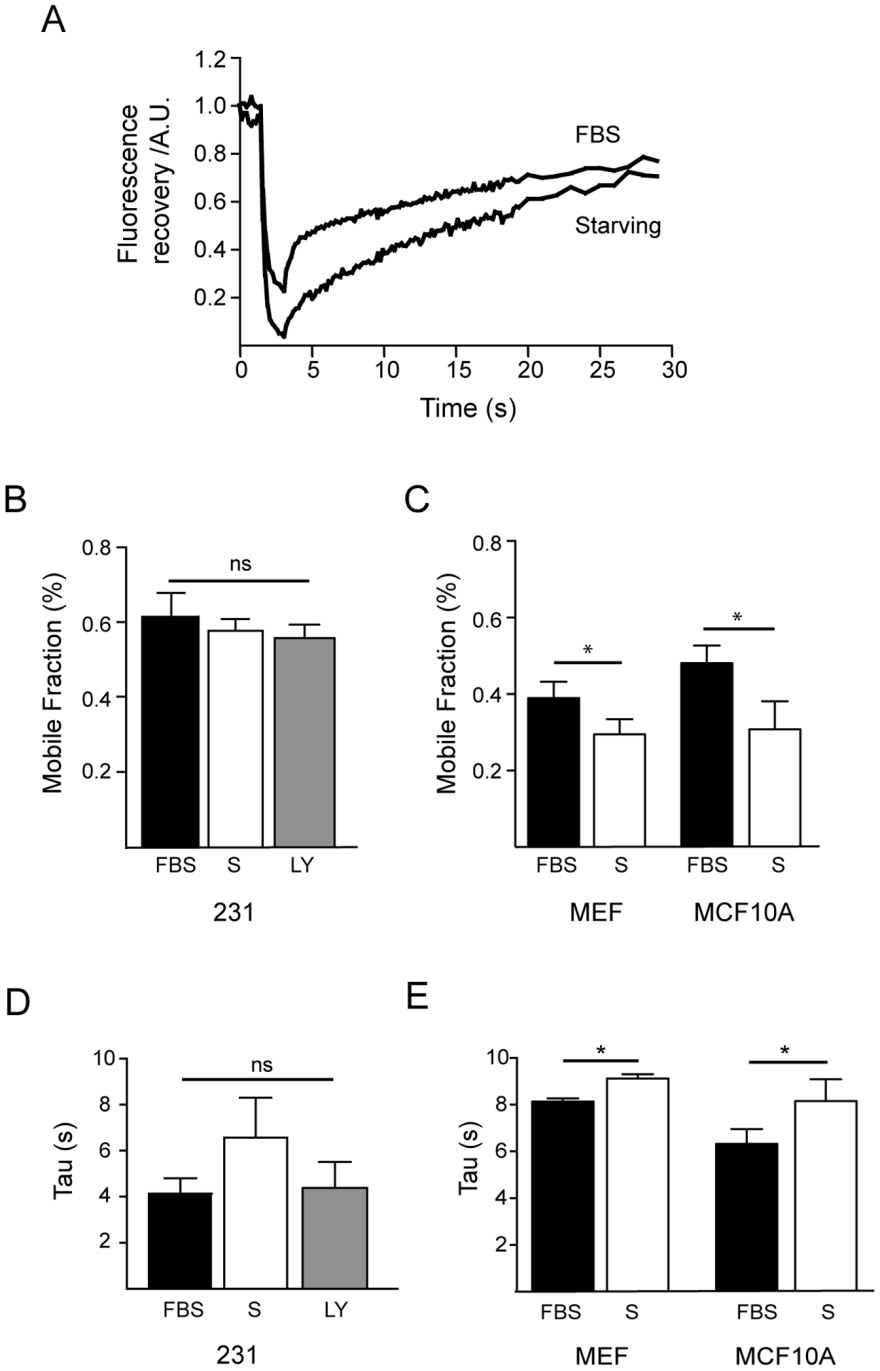
Actin recovery is independent of the presence of extracellular growth factors. A) Example of fluorescence recovery after FRAP under control conditions (10% FBS, FBS plot) and serum starvation for 16 hours (Starving plot). B) Summary data of the mean mobile fraction in MDA-MB-231 cells. The mean mobile fraction under FBS conditions was 62±5.2% (n = 6), as compared to 58±3% after 16 hours of starvation and 57±2% (n = 6) after PI3K inhibition with LY294002 (n = 6). It should be noted that we have included new control cells, growing in parallel and analyzed on the same day and under the same conditions; therefore, the average mobile fraction has a slightly different value that the overall mean average (70% versus 65%). C) The mobile fraction of non-cancerous cells under serum-starved conditions was reduced to 27±5.2% (n = 10) in mouse fibroblast and to 28±2.4% in MCF10A (n = 6), (p<0.05; Student's t-test). D) Recovery time summary graph. MDA-MB-231 growing in FBS tended to show faster dynamics (4.14±0.6 s) not statistically significant from that of starved cells (6.6±1.7 s). The use of LY294002, a chemical inhibitor of PI3K, did not affect recovery time (4.2±1.0 s). E) In contrast, serum starvation affected the recovery time of non-cancerous cells, increasing the time constant from 8.2±0.2 s to 9.5±0.1 s for MEFs (p<0.05) and 6.5±0.4 s to 8.0±1.2 s for MCF10A cells (p<0.05; Student's t-test).

Acquired independence from extracellular growth factor signaling is a typical characteristic of breast cancer cells [51]. In non-cancer cell lines, cell motility and polarization are led by a local accumulation of PIP_3_; mostly driven by PI3K activation or local integrin activation. In fact, aberrant PI3K signaling is a recurring theme in both the initiation and progression of a variety of cancers, including breast cancer [45]. We next wanted to test the dependence of actin dynamics at the leading edge on the extracellular presence of growth factors. To this end, we studied the actin dynamics of cancer and non-cancer cell lines after a 16-hour serum starvation period. The results indicate that MDA-MB-231 mobile fraction was unaffected by the serum starvation condition (Figure 4A), with an average mobile fraction value of 58±3%, and a mean tau of recovery of 6.6 seconds, which was not statistically different from the values obtained in the presence of serum (Figure 4B and D). One immediate consequence of the absence of growth factors is the reduction of PIP_3_ signaling at the membrane level. To confirm the influence of PIP_3_ on actin modulation, actin dynamics at the leading edge was analyzed 30 minutes after having treated the cells with the PI3K inhibitor LY294002 (20 µM). The mobile fraction showed a mean value of 57±2%, and the fluorescence recovered with a time constant of 4.2 seconds, which was not statistically different from that under control or serum starvation conditions (Figure 4B and D).

In contrast, mobile fraction of MCF10A cells was reduced under serum starvation conditions (mean value of 28±2.4% compared to 51% under control conditions; Figure 4C). Similar dependence of extracellular serum was observed in MEFs, whose mobile fraction declined from a value close to 40% to a value around 27% (Figure 4C). Recovery time was also affected, in MCF10A cells increased from 6.5±0.4 seconds to 8.0±1.2 seconds, while in MEF cells increased from 8.2±0.2 to 9.5±0.1 seconds (Figure 4E).

From the results obtained in this section, we conclude that in MDA-MB-231 cells, both the actin mobile fraction and the time course of recovery are unaffected by the presence of extracellular growth factors and membrane regulation of PIP_3_ levels.

### PfnI up-regulation increases cell size and the number of FAs

Several actin binding proteins are deregulated in cancer cell lines [28], [52], among them Profilin (PfnI), which was proposed as a tumor suppressor protein [27], [30]. PfnI expression levels are significantly down-regulated in various types of adenocarcinoma cells. As stated, silencing PfnI expression in MDA-MB-231 cells leads to increased motility and invasiveness [32], while conversely, PfnI up-regulation restores a non-tumorigenic phenotype.

**Figure 5:**
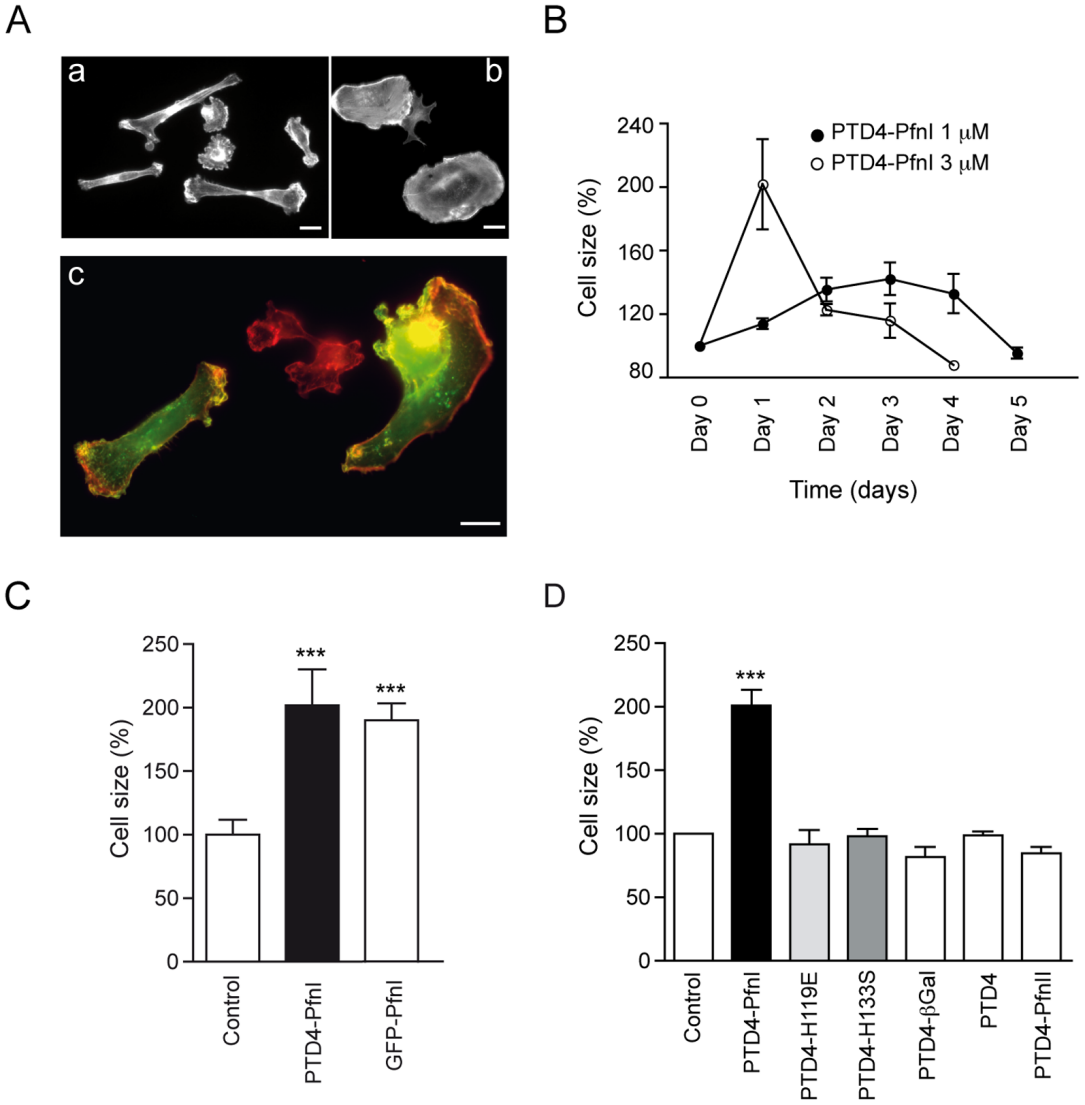
Increased Profilin I intracellular levels induce cell spreading. A) MDA-MB-231 control cells (a) and treated with 3 µM of PTD4-PfnI for 24 hours (b). Bottom picture: MDA-MB-231 control cells (red) and cells transfected with GFP-PfnI (green) (c). Notice how transfected cells displayed a large cell size. B) Temporal evolution in cell size of a culture treated with either a chronic application of 1 µM of PTD4-PfnI (every day, n = 430) or a single application of 3 µM (n = 540). C) Summary of the average changes in cell size after treatment with a transduction version of Profilin I (PTD4-PfnI I, 3 µM, n = 230) or after transfection with GFP-PfnI (n = 340). On average cell area was larger when compared with the control MDA-MB-231 cells (p<0.005; Student's t-test). D) Summary of the effects of different transduction proteins: Point mutations of Profilin (H119E and H133S), PTD4-ßGal, PTD4 (a transduction domain without any protein attached) and PTD4-PfnII (the neuronal isoform). None of the treatments altered MDA-MB-231 area (n>100, Student's t-test). Cells were visualized either with the help of Oregon green-Phalloidin or Phalloidin Alexa Fluor-594.

To study the relationship between PfnI and actin dynamics in cancer cell lines, we have used a recombinant version of PfnI (PTD4-PfnI). This fusion protein can cross biological membranes and accumulates in the cytoplasm, inducing intracellular actin polymerization and the formation of ectopic lamellae [24], [40]. Protein transduction is a highly efficient technique, reaching all cultured cells in a matter of minutes. It has been reported that the increase in PfnI intracellular levels in certain types of cancer expands cell size and reduces motility [30], [32]. To validate our recombinant protein, we first studied the effect of PTD4-PfnI on MDA-MB-231 cell morphology (Figure 5Aa–b). To this end, culture cells were treated either chronically, adding 1 µM of PTD4-PfnI every day for four days or with a single application of 3 µM (Figure 5B). The level of intracellular PTD4-PfnI reached in both conditions was estimated following a protocol described in Syriani et al., 2011 [24]. On average, after a chronic treatment, the relative amount of PfnI raises a 8±3.3%, while after a single application of PTD4-PfnI levels increased a 12±8.2% (variation between 2% to 22%, data not shown). In line with previously published data, a rise in PfnI intracellular levels caused a transient, concentration-dependent increase in cell area (Figure 5B and C). During the chronic application (closed circles, Figure 5B), the cells progressively spread out reaching a maximum value of 140% after 4 days (day 4) and, after the removal of PTD4-PfnI from the culture media, their size returned to the control values (day 5). Similarly, a single application of 3 µM of PTD4-PfnI (open circles, Figure 5A) induced a 200% cellular spreading (Figure 5C) followed by a progressive reduction in cell size thereafter (days 0 to 2; Figure 5B and C). In order to confirm the effects of PfnI on cell size, cell spreading was also quantified in GFP-PfnI-transfected MDA-MB-231 cells (Figure 5Ac). On average, transfected cells underwent an expansion of cell area that was close to 200%, a value in the same range as that obtained with the 3 µM PTD4-PfnI treatment (Figure 5C). Thus, we can conclude that increasing PfnI levels, either by transduction or by overexpression, induced similar changes in MDA-MB-231 cells morphology.

To confirm that both actin and the polyproline binding domain of PfnI are necessary for cell size increase, two recombinant proteins were generated. We fused PTD4 with H119E [53], a mutated form of Profilin with reduced affinity for actin, and also with H133S, a mutation in the polyproline domain [54]. Neither of the mutations affected cell size, confirming that functional actin and polyproline domains were necessary for the cell size increase to occur; therefore corroborating the ability of PTD4-PfnI to effectively modify the intracellular concentration of PfnI (Figure 5D). As a control for possible non-specific effects, a recombinant protein PTD4-β-galactosidase and the transduction domain alone (PTD4) were also tested (Figure 5D). Finally, a recombinant protein fusing the transduction domain PTD4 with Profilin II (PfnII) (the neuronal isoform) was also studied; the absence of effect on cell size suggests that PfnII is not a viable substitute for PfnI (Figure 5D).

The cell size increase prompted us to quantify the mean number of focal adhesions (FA) per cell (Figure S2). To this end, we identified FA by means of vinculin staining. Along with the increase in cell size, PTD4-PfnI treated cells almost doubled the number of vinculin-positive FA (mean value of 18±0.1 versus 11±0.09 FA/μm^2^). No statistical differences were evident in FA size in terms of either distribution area or mean area as the result of the treatment (2.3 µm^2^ under control conditions and 2.4 µm^2^ after PTD4-PfnI treatment; supporting data 2B–C). All together, these results indicate that PTD4-PfnI can successfully modify intracellular Profilin concentrations, affecting cytoskeleton dynamics.

### PfnI overexpression regulates actin dynamics at the leading edge

Profilin I plays an important role in promoting the exchange of ADP for ATP on G-actin, and funneling actin polymerization by recruiting actin monomers to the open barbed end. Therefore, our next question was regarding how actin polymerization at the leading edge would be affected by intracellular PfnI concentrations.

**Figure 6:**
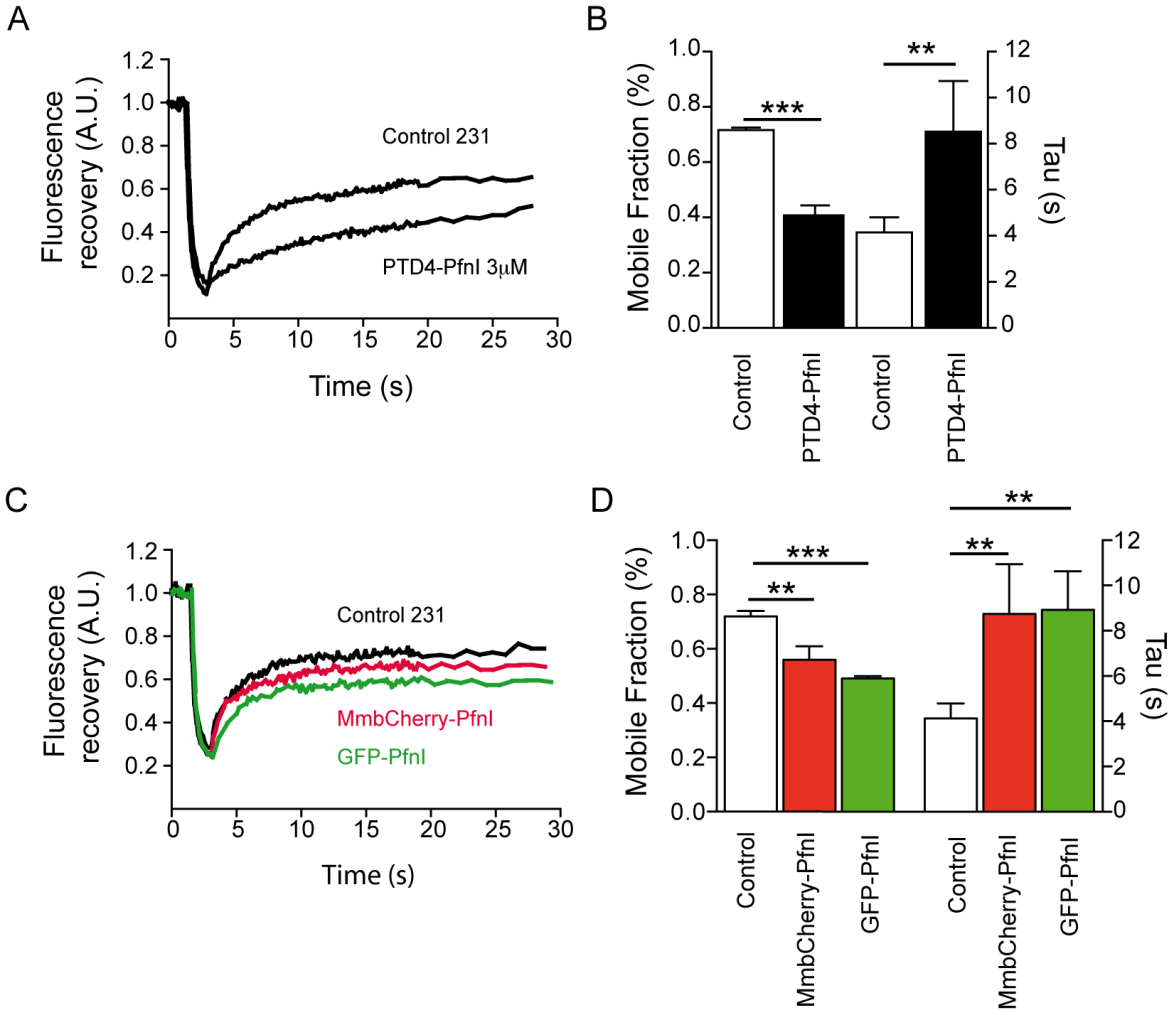
Profilin intracellular levels modify actin treadmilling dynamics. A) Example of fluorescence recovery in a control MDA-MB-231 cell and after a 24 h treatment with PTD4-PfnI 3 µM. B) Plot summary of the average mobile fraction (left axis) and mean tau (right axis) values before (white box) and after PTD4-PfnI treatment (black box; n = 24). PTD4-PfnI caused a 29% reduction of the mobile fraction from 69±1% to 40±2% and increased the recovery time to a mean value of 8.2±2.2 s (p<0.05 and p<0.005; Student's t-test) C) Example of actin fluorescence recovery in a MDA-MB-231 cell transfected with GFP-PfnI (green) or MembraneCherry-PfnI (red) showing a similar effect to that of PTD4-PfnI treatment. D) Summary plot of the average mobile fraction (left axis) and mean recovery time (right axis). On average, transfection with GFP-PfnI reduced the mobile fraction to a mean value of 50±1.2% (n = 15), while it increased the time course of recovery to a mean value of 9.0±2.2 s. MmbCherry-PfnI expression reduced mobile fraction to 58±3% and slowed recovery time to 8.9±2.2 s (n = 15). (p<0.05 and p<0.005; Student's t-test).

To this end, FRAP experiments were performed with the stable GFP-actin transfected cell line, treated with 3 µM PTD4-PfnI for 24 hours. Under these conditions, the actin mobile fraction decreased from a mean value of 69±1 to 40±2% (Figure 6A–B), the treatment also doubled the recovery time from 4.14±0.6 to 8.2±2.2 seconds (Figure 6A–B).

These results were confirmed by experiments in which PfnI levels were elevated by transfection. The GFP-PfnI expresser cell line was transiently transfected with actin-cherry to visualize actin at the leading edge. In these cells, the mobile fraction was reduced to a value of 50±1.2%, a range similar to that observed after PTD4-PfnI treatment. Recovery time was slower that controls with a tau of 9 seconds (Figure 6C-D).

Membrane targeting of PfnI has been reported as mediator of PfnI effects, in fact the PfnI sequence contains two PIP binding domains [15], [55]. To address the specific effect of membrane localization of PfnI on actin polymerization, we generated a stable MDA-MB-231 cell line expressing membrane-Cherry-PfnI (MmbCherry-PfnI). The membrane targeting domain, fused to the fluorescence protein, efficiently directs all protein expression to the membrane. The level of MmbCherry-PfnI overexpression was close to 9% of total PfnI expression (data not shown). To confirm the biological activity of the recombinant protein, changes in cell area were analyzed [32]. On average, cell area increased to 225% (data not shown), similar to the results obtained with GFP-PfnI expresser cells or PTD4-PfnI treated cells. To analyze actin dynamics, GFP-actin stable transfected cells were transiently transfected with MmbCherry-PfnI. Under these conditions the actin mobile fraction decreased to 58±0.3% and the recovery time slowed down to 8.9±2.2 s (Figure 6C–D). Overall, these results were consistent with an increase in filament population and a reduction in the actin polymerization rate following the increase of PfnI intracellular levels.

### Increasing the PfnI concentration negatively regulates cell movement

**Figure 7:**
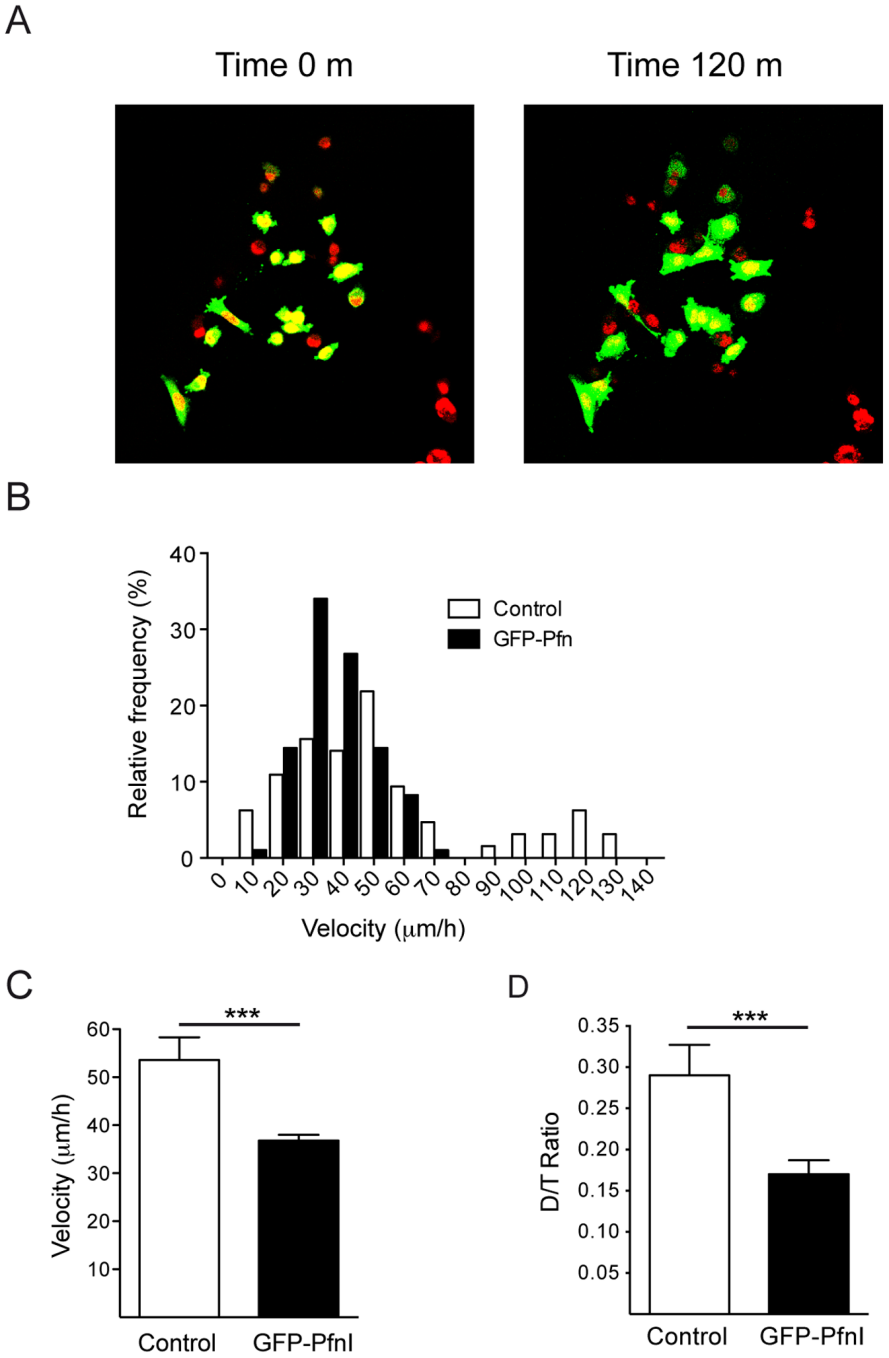
Profilin overexpression reduces cell velocity and directionality. A) GFP-PfnI transfected cells (green) were mixed with control non-transfected MDA-MB-231 cells, and the nuclei were stained with Draq5 nuclear vital dye (red). Cultures were visualized for 8 hours; the images were two examples taken at the beginning of the experiment (time 0 m) and after two hours (120 m). Notice that the same sample contains transfected and non-transfected cells. Fluorescence levels are oversaturated to facilitate visualization. B) Individual cell velocity distribution. GFP-PfnI transfected cells displayed lower velocities (black columns) while control cells displayed a broad distribution (white columns), with a low velocity population (between 10 and 70 µm/h) and a clear second population of faster cells with velocities between 90 and 130 µm/h. C) Plot summaries of the mean velocity. Control cells and GFP-PfnI transfected cells have an average velocity of 53.5±5.5 µm/h and 36. 7±0.3 µm/h, respectively D) Mean values of the D/T ratio comparing control (0.29±0.03) and after GFP-PfnI transfecction (0.17±0.01) (p<0.005; Student's t-test).

Lamellipodial protrusions initiate and define the direction of cell movement. The reduced actin dynamics at the leading edge is suggestive of impaired cellular motility. To study the relation between motility and actin dynamics, we employed a MDA-MB-231 GFP-PfnI stable transfected cell line. The velocity and directionality of individual GFP-PfnI transfected cells were estimated and compared to those of non-transfected cells [42]. The use of transfected cells instead of a PTD4-PfnI is advantageous as it allows both the control and the GFP-PfnI treatment to be cultured on the same plate. Accordingly, cells were stained with the nuclear-vital dye DraQ-5 and the individual cell tracks were followed for 8 hours (Figure 7A). The frequency distribution of individual velocities indicates the presence of two populations of control cells, a first population characterized by a faster velocity (10 to 70 µm/h) and a second one with velocity between 90 to 130 µm/h (Figure 7B, white bars). In contrast, the velocity distribution of GFP-PfnI transfected cells shows only a single population of cells with lower velocities. The mean cell velocity was 53.5±5.5 µm/h for the control cells and 36.7±0.3 µm/h for the GFP-PfnI transfected cells (Figure 7B–C). Changes in cell movement directionality have been associated with changes in Rac1 activation and cell migration chemotaxis [42]. PfnI intracellular levels also affected directionality of movement. The percentage of linear movement of each individual cell was calculated by dividing the estimated linear distance between the starting and the end point (D) during the time elapsed by the total distance travelled by the cell (T). Control cells had an average D/T value of 0.29±0.03, as compared to 0.17±0.01 in the case of GFP-PfnI transfected cells (Figure 7D). Thus, PfnI transfection reduces cell velocity and promotes more random displacement.

## Discussion

In this report, we used FRAP technique to compare actin turnover at the cell leading edge in cancerous and non-cancerous cells. Our results indicate that (1) The tumor cell lines examined present a high motility actin; (2) MDA-MB-231 actin dynamics at the leading edge are independent of extracellular growth factors; and (3) Profilin I negatively regulates actin polymerization, reducing actin mobile fraction and slowing the recovery time.

Actin dynamics are an integral part of cell migration. Elevated actin dynamics at the leading edge were previously described in the murine melanoma B16-F1 cell line [39]. In this cell type, actin fluorescence recovers with values close to 60% after 30–40 seconds, parallel to a series of actin-binding proteins, such as cortactin, Arp2 and Abi, which show similar recovery times [39]. In clear contrast, non-cancerous cells are characterized by a higher immobile fraction of actin and a slower turnover, i.e., they have a more stable actin cytoskeleton and slower treadmilling. Our analysis is limited to a few cell types of epithelial origin, thus we cannot generalize with respect to all cancer cell types, nevertheless the results are suggestive of a different mechanism of actin regulation at the leading edge between cancer and non-cancer cells. Further experiments analyzing cancer cells of other origins will be needed to extend our conclusions.

Both cell lines tested (MCF10A and MEF), despite the different origins, evidenced a lower mobile fraction, and their actin cytoskeleton at the leading edge was regulated by the presence of serum in the culture media. Actin dynamics and directional cell movement are highly dependent on phosphoinositide membrane levels [42]. The activation of PI3K through the tyrosine-kinase receptors and integrin pathways promotes PIP_3_ formation that, in turn, facilitates the membrane targeting of several proteins implicated in actin dynamics, such as Tiam (a major Rac1 GEF), Ena-VASP and Profilin [15], [17], [34], [36], [38]. Accordingly, serum-starved fibroblasts do not recover after FRAP, suggesting a severe inhibition of actin treadmilling. In our experiments, we failed to observe any differences in the mobile fraction of MDA-MB-231, meaning that the population of filaments is undergoing a continuous treadmilling, independent of external clues. The only effect of the lack of serum was an increase in recovery time, suggestive of a slow rate of actin polymerization. Several factors can account for this phenomena; for instance, membrane targeting of the anti-capping protein ENA/VASP modulates the assembly rate of the actin network, filament length and membrane protrusion. However, even with a total lack of ENA/VASP, membranes were still able to protrude and cells did not lose their motility [56]. Thus, by regulating the levels of anti-capping proteins, actin dynamics can be modulated without affecting nucleation [57].

Our results suggest that the rapid incorporation of actin at the leading edge is a hallmark of cells with high motility. It is tempting to speculate on the relationship between actin dynamics and motility. When the velocity of individual MDA-MB-231 cells was analyzed, the linear velocity was higher in control cells, compared to GFP-PfnI transfected cells (50 versus 30 µm/h). These results are in agreement with those of other authors, reflecting that an increase in PfnI levels reduces cell velocity and favors random movements [32].

Cellular velocity depends on several factors, among which lamella stability is one of the most important. Counterintuitively, a slow protruding lamella allows faster cell motility by providing a more stable anchor to the substrate in 2D culture conditions [36]. In contrast, cancer cells are characterized by a rapid lamella protrusion. Further experiments analyzing the relationship between the half-life of the focal complex and lamella velocity would clarify this point.

Profilin I is an ubiquitously expressed actin-binding protein, required for cell migration and polymerization funneling. PfnI has been regarded as a tumor-suppressor, due to the fact that its levels are constitutively down-regulated in breast and other cancer cell lines [27], [30]. Its role in promoting actin polymerization, membrane protrusion and motility has been well established [12], [58], [59]. However, in several tumor cells, PfnI reduction has been determined to enhance motility and invasiveness [32], [38]. In contrast, even a moderate overexpression of PfnI induces actin stress-fiber formation, up-regulates focal adhesion, promotes cell spreading and impairs motility and invasiveness [27], [29], [32], [60]. In MDA-MB-231 cells, the up-regulation of PfnI reverts to an epithelial phenotype [30], increases cellular spreading and focal adhesion number, raises F-actin levels [32] and restores adhesion junctions [60]. Interestingly, PTD4-PfnI increases cellular spreading, while the neuronal isoform, PfnII, was not able to induce similar effects. PfnI and II bind different ligands with high specificity [55], [61], the PLP binding domain targets Pfn to their ligands. Crystalographic structures of PfnI and II indicate that both structures can overlap, however charge distribution is quite different and this affects their binding affinity for various ligands. This might explain why PnfI and PfnII, despite having functional redundancy, have different roles in actin-dependent process [59]. As it was recently demonstrated in tumor cell migration in a breast cancer cell model, PfnII preferentially drives actin polymerization by an Ena/Vasp Like protein interaction mechanism [62].

In the present study, we evaluate how PfnI affects actin polymerization at the leading edge. Our results showing a decrease in mobile fraction are consistent with the previous observations following PfnI overexpression. The increase in F-actin levels and the induction of a more stable cortical rim of actin would result in a lower mobile fraction. Fluorescence recovery was also affected, indicating a slower rate of actin polymerization after raising Pfn levels.

What are the potential molecular mechanisms underlying this effect? Recently, it has been proposed that PfnI can act as a regulator of PIP availability. In this sense, overexpression of PfnI would reduce PI(3,4)P_2_ at the membrane level, which in turn would prevent the membrane targeting of the anti-capping lamellipodin-Ena/VASP complex to the leading edge [34], [38]. PfnI has two phosphoinositide binding sites that allow the protein to be located at the membrane [15]. The main role of the phosphoinositide binding site could be to recruit PfnI to the membrane, close to the sites where polymerization is taking place. As suggested, the increase in Profilin levels within the membrane would limit the availability of PIP_2_, concomitantly decreasing the levels of Ena/VASP at the leading edge [34], [38]. Ena/VASP is considered a filament elongator that enhances F-actin polymerization *in vitro* [57], [63]. We hypothesized that the imbalance in PIP levels, induced by the high levels of PfnI, would favor the binding of CapZ to F-actin filaments. As demonstrated, increasing the PIP_2_ concentration causes a rapid dissociation of CapZ from F-actin [2]. Our analysis of actin dynamics supports this model. In parallel, raising the intracellular levels of PfnI would reduce the concentration of Ena/VASP at the membrane level, which should slow down filament elongation rate. Interestingly, membraneCherry-PfnI transfection has similar effects on actin dynamics as GFP-PfnI or PTD4-Pfn I treatments, reinforcing the idea that the localization of PfnI at the membrane level is crucial for its function.

## Supporting Information

Figure S1 **Cells transfected with monomeric GFP display a fast recovery after photobleaching.** A rectangular area of 2×4 µm was photobleached. The graph displays two examples of recovery. Time courses were best-fitted by a monoexponential curve with values between 100–200 ms. Under these conditions, the recovery of fluorescence was driven only by GFP diffusion.
(TIF)

Figure S2 **Increasing Profilin concentration up-regulates the number of focal adhesions.** A) Vinculin was localized at focal adhesions and in the focal complexes. Top picture: overlap composition of MDA-MB-231 cells stained for actin fibers (green) and viculin (red). B) Distribution of FA sizes before (black column) and after (open columns) PTD4-PfnI treatment (3 µM for 24 h). No differences in distribution were found. C) Total FA number (left axis) and mean area (right axis), respectively (Student's t-test).
(TIF)
