# Supplementary figures and images for: Actin Filaments at the Leading Edge of Cancer Cells Are Characterized by a High Mobile Fraction and Turnover Regulation by Profilin I
Source: PLoS One. 2014 Jan 17;9(1):e85817. doi: 10.1371/journal.pone.0085817 (PMC3895011; doi:10.1371/journal.pone.0085817)

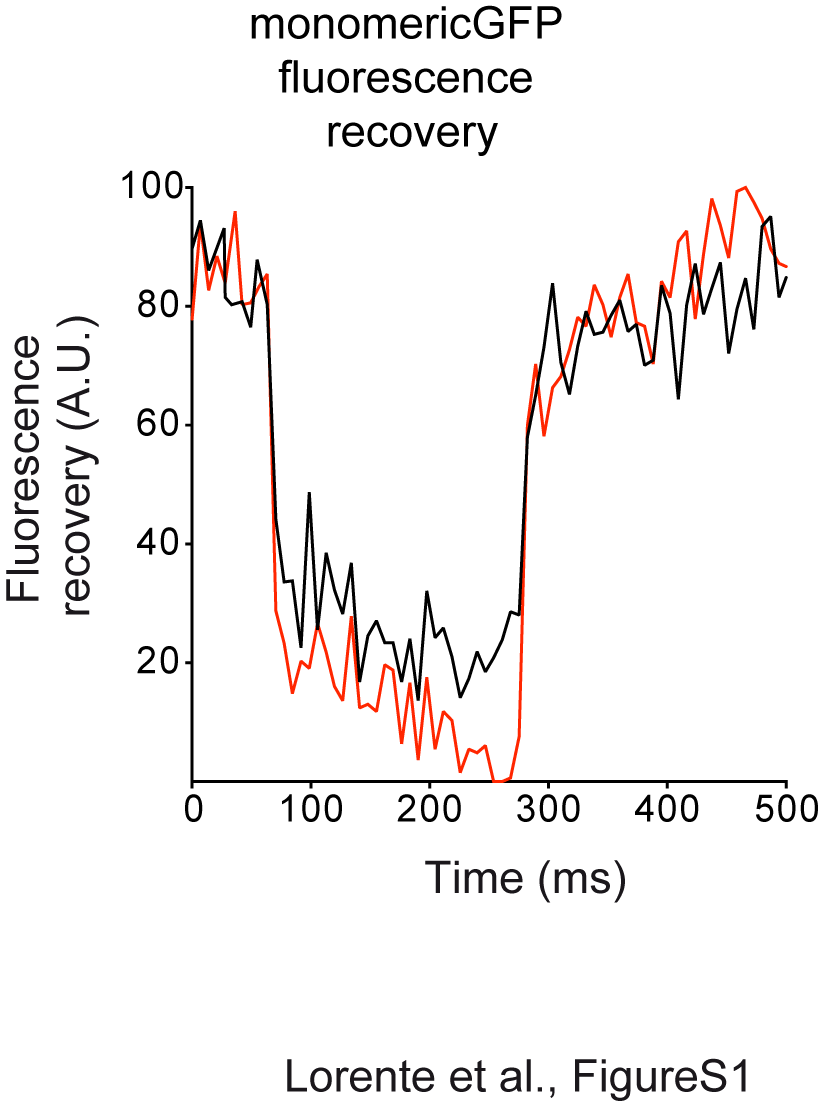

Supplement: Figure S1 — Cells transfected with monomeric GFP display a fast recovery after photobleaching. A rectangular area of 2×4 µm was photobleached. The graph displays two examples of recovery. Time courses were best-fitted by a monoexponential curve with values between 100–200 ms. Under these conditions, the recovery of fluorescence was driven only by GFP diffusion. (TIF) [file pone.0085817.s001.tif]

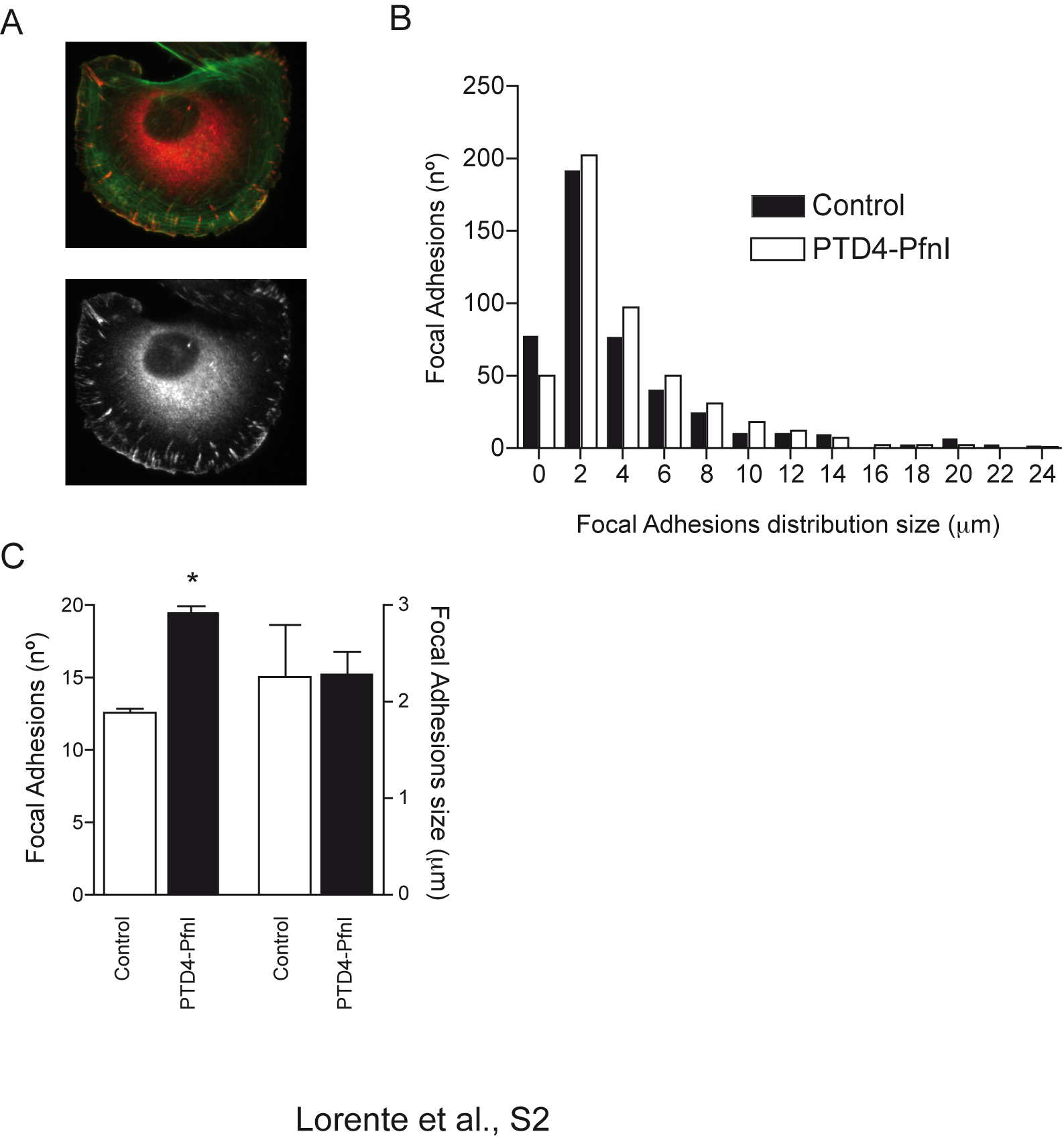

Supplement: Figure S2 — Increasing Profilin concentration up-regulates the number of focal adhesions. A) Vinculin was localized at focal adhesions and in the focal complexes. Top picture: overlap composition of MDA-MB-231 cells stained for actin fibers (green) and viculin (red). B) Distribution of FA sizes before (black column) and after (open columns) PTD4-PfnI treatment (3 µM for 24 h). No differences in distribution were found. C) Total FA number (left axis) and mean area (right axis), respectively (Student's t-test). (TIF) [file pone.0085817.s002.tif]
